# Exploring the feedback limits of quantum dot lasers for isolator-free photonic integrated circuits

**DOI:** 10.1038/s41377-026-02185-w

**Published:** 2026-01-30

**Authors:** Ying Shi, Bozhang Dong, Xiangpeng Ou, Artem Prokoshin, Chen Shang, John E. Bowers, Yating Wan

**Affiliations:** 1 Integrated Photonics Lab, King Abdullah University of Science and Technology (KAUST), Thuwal, 23955-6900 Kingdom of Saudi Arabia; 2 Institute for Energy Efficiency, University of California Santa Barbara, Santa Barbara, CA 93106 USA

**Keywords:** Semiconductor lasers, Quantum dots, Integrated optics

## Abstract

Reflections from on-chip components pose significant challenges to stable laser operation in photonic integrated circuits (PICs). Quantum dot (QD) lasers, with low linewidth enhancement factors and high damping rates, are promising for isolator-free integration, yet earlier feedback studies were capped near −10 dB feedback and never reached coherence collapse (CC). As a result, one could only conclude that QD lasers tolerate feedback up to –10 dB, leaving open whether they remain reliable in practical PICs where lower coupling losses allow much stronger feedback. Here, we optimized QD lasers through advanced epitaxial growth and fabrication and developed a setup that delivers feedback up to 0 dB. Under these conditions, we observed CC at −6.7 dB (21.4% feedback), extending the feedback tolerance by tens of decibels beyond quantum-well (QW) lasers. We further demonstrated penalty-free 10 Gbps operation, robust thermal stability with ±0.5 dB drift across 15–45 °C, >100 h continuous testing, and ~±0.3 dB reproducibility across devices. Modeling indicates even stronger tolerance in realistic PIC cavities, and benchmarking shows our device rivals hybrid DFB–resonator platforms while outperforming other QW, QD, and VCSEL lasers. Together, this work provides the most comprehensive assessment of QD laser feedback tolerance to date and establishes practical design rules for isolator-free PICs.

## Abstract (web summary)

By accessing the coherence-collapse regime under strong on-chip feedback, quantum dot lasers demonstrate unprecedented robustness, supporting penalty-free operation and practical isolator-free photonic integrated circuits.

## Graphical abstract

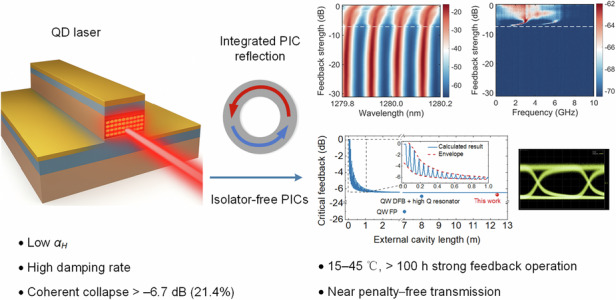

## Introduction

The integration of laser sources into photonic integrated circuits (PICs) represents a crucial step toward scalable, energy-efficient optical systems^1–3^. Yet, stable on-chip laser operation is often hampered by optical feedback stemming from unavoidable reflections within the PIC. Such reflections originate from both coherent sources, including grating couplers, material interface transitions, and externally connected optical fibers^4,5^, as well as incoherent sources, notably amplified spontaneous emission (ASE) noise within active components like semiconductor optical amplifiers (SOAs), modulators, or photodetectors. These parasitic reflections can destabilize conventional on-chip lasers, inducing performance degradations ranging from intensity noise and mode instability to the extreme state of coherence collapse (CC), where the laser output becomes chaotic and unusable.

Currently, III-V quantum well (QW)-based distributed feedback (DFB) lasers dominate optical communication networks but exhibit high sensitivity to optical feedback. Feedback levels as modest as -30 dB can severely impair their performance^6,7^. To mitigate this, isolators are commonly employed, but their inclusion increases system complexity, cost, and footprint, countering the advantages of integration. Recent studies addressed the issue of CC by incorporating an on-chip high-Q resonator, however, at the price of larger footprint, higher operation complexity and higher cost^8^. The emergence of quantum dot (QD) lasers marks a turning point in addressing these challenges. Unlike bulk or QWs active regions, QDs confine carriers in three dimensions, enhancing photon-electron interactions and enabling superior device metrics of lower threshold currents^9^, improved thermal stability^10,11^, and enhanced tolerance to defects and remarkable noise properties^12^. For large-scale PIC integration, QD laser platform is compatible with both monolithic and heterogeneous integration. We have demonstrated electrically pumped QD lasers monolithically grown on 300 mm Si wafers^13^ and, in a heterogeneous scheme, QD lasers evanescently coupled to silicon waveguides^14^—an approach recently followed by Intel in their 300 mm silicon photonics foundry^15^. Crucially, QD lasers exhibit near zero linewidth enhancement factors (*α*_*H*_)^16^, high damping rates^17^, and ultrafast carrier dynamics, enabling Class A-like behavior and exceptional resilience to external perturbations such as optical feedback and injection^18^. Recent demonstrations have reported *α*_*H*_ as low as 0.13, enabling penalty-free 10 Gbps transmission under −7.4 dB feedback^7^ and isolator-free 128 Gbps operation under −13.0 dB feedback, far exceeding the feedback tolerance of traditional QW DFB lasers. Additionally, controlling the ratio of excited state (ES) to ground state (GS) threshold currents can further stabilize the laser and mitigate complex dynamics in the ES regime^19,20^.

Despite these promising indicators, understanding of QD laser feedback tolerance has been limited by practical experimental constraints. Previous studies of QD Fabry–Perot (FP) lasers were limited to about -10 dB feedback due to chip-to-fiber coupling losses^7,17,21,22^. Since no CC was observed under those conditions, QD lasers were sometimes viewed as nearly immune to feedback. However, this does not represent on-chip realities. Integrated PICs with lower coupling losses can face much stronger reflections, for example, in on-chip optical sensors, LiDAR arrays with reflective surfaces^23^, or PICs with integrated SOAs and other reflective elements. Such scenarios could push even robust QD lasers toward their true feedback tolerance limits and raise a clear question: can QD lasers operate reliably without isolators in real PICs?

To answer this, we optimized epitaxial growth and fabrication of QD FP lasers, achieving a threshold current of 11.7 mA (159 A/cm^2^), a maximum output power of 102 mW, and a large ES-to-GS lasing threshold ratio of 21. Nearly all prior feedback studies employed ridge-waveguide FP cavities; using the same ensures direct, apples-to-apples benchmarking and isolating effects of the QD gain medium itself. In contrast, distributed-feedback (DFB) or distributed-Bragg-reflector lasers rarely align their Bragg wavelengths with the material gain peak, which raises the effective *α*_*H*_ from ~0.5 to 2–3^12^ and interferes with assessing the feedback tolerance. Focusing on FP devices therefore probes the intrinsic limit of the QD gain medium rather than effects introduced by cavity design.

We then developed a specialized setup that delivers feedback up to 0 dB, including an in-loop SOA to overcome passive loss. With this platform, we systematically studied laser behaviors from weak to extremely strong feedback, combining optical and electrical spectra, relative intensity noise (RIN), and data transmission performance. We identified a high CC threshold of -6.7 dB, demonstrated penalty-free 10 Gbps data rates transmission under external modulation near the threshold, and preserved open eyes as feedback approaches 0 dB. These results remained stable across 15–45 °C (±0.5 dB drift), for more than 100 h of operation, and across multiple devices (~±0.3 dB).

Lang–Kobayashi modeling further supports these findings, predicting that centimeter-scale cavities typical of integrated layouts shift the CC boundary closer to 0 dB, so QD lasers are most tolerant where they will actually be deployed. Benchmarking shows that our standalone device at −6.7 dB is comparable to the best hybrid DFB plus resonator platforms and is substantially more tolerant than representative QW, QD, QDash, vertical-cavity surface-emitting laser (VCSEL) devices. In summary, this work provides the first direct measurement of the CC threshold in QD lasers under realistic feedback and connects it to system performance, stability, reproducibility, and on-chip modeling, establishing clear guidelines for isolator‑free photonic integration.

## Results

### Quantum dot laser fabrication and characteristics

**Fig. 1:**
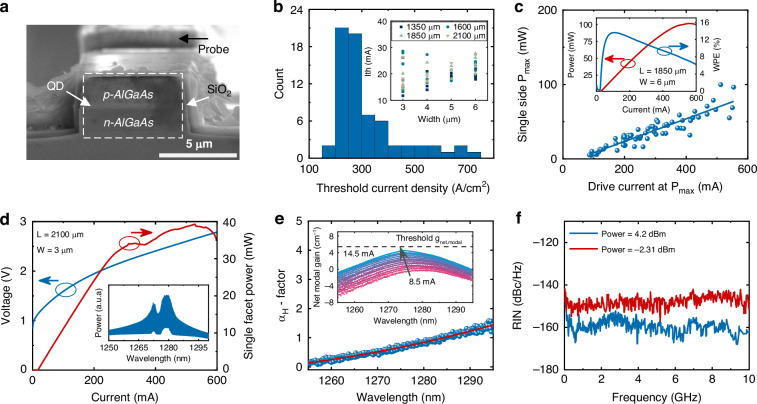
Static QD laser performance characterization. **a** Cross-sectional SEM of the device structure. **b** Histogram of *J*_th_ for all tested lasers, with the majority falling between 200 and 300 A/cm². **c** Maximum single-side GS CW lasing output power versus drive current; The dashed line represents the best fit with a slope of 0.152 W/A. Inset shows L-I-V characteristics and the wall-plug efficiency (WPE) of a laser achieving 102 mW maximum single-side GS power. **d** L-I-V characteristics and emission spectrum at 3 × *I*_th_ bias of a device used in the subsequent feedback dynamic measurements. **e** Measured *α*_*H*_ as a function of wavelength. The inset shows net modal gain at various subthreshold currents. **f** RIN spectra at two power levels

The QD laser structure was grown on a (001) GaAs wafer using a Veeco Gen-II solid source molecular beam epitaxy (MBE) system (Materials). The as-grown wafer was then processed into deeply etched ridge-waveguide lasers using standard semiconductor dry etching and metallization techniques. Although etching through the active region is known to accelerate degradation in QW lasers—sidewall defects introduce non-radiative recombination centers—the situation is fundamentally different for QD devices. Three-dimensional carrier confinement keeps carriers localized away from etched surfaces, lowering the surface-recombination velocity and contributing to the reliability. Long-term ageing tests on comparable deeply etched InAs/GaAs QD ridge lasers grown on Si project median lifetimes >22 years at 80 °C after 1200 h of constant-current stress^24^, confirming that reliability is not compromised. Deep etching also provides a high lateral refractive-index contrast that tightens optical confinement, and suppresses the linewidth-enhancement factor, which directly underpins the exceptional feedback immunity demonstrated later. Figure 1a shows the cross-sectional SEM image of the device structure. A Ti/Pt/Au *p*-contact was deposited on top of the ridge mesa, while a Ni/Ge/Au/Ni/Au *n*-contact was deposited on the exposed *n*-contact layer. The device was passivated with a 5-nm atomic-layer deposited (ALD) Al_2_O_3_ followed by 500 nm plasma enhanced chemical vapor deposition (PECVD) SiO_2_. After opening vias to the *p* and *n* contact, Ti/Au probe metal were deposited. The cleaved laser bars were mounted on the copper heat sink for continuous-wave operation and characterization.

Figure 1b depicts a histogram of the threshold current density (*J*_th_) of all measured QD lasers, with 60% of the total devices falling between 200 and 300 A/cm^2^ (40–60 A/cm^2^ per QD layer). The inset shows the threshold current (*I*_th_) for all lasers with varying ridge widths and lengths. The lowest *I*_th_ is 11.7 mA for a 1350 × 3 μm^2^ cavity, while the lowest *J*_th_ is 159 A/cm^2^ for a 2100 × 6 μm^2^ cavity, which corresponds to 32 A/cm^2^ per QD layer. Figure 1c plots the maximum single-side CW lasing output power (GS) versus drive current for all devices. A best fit slope of approximately 0.152 W/A serves as a conservative estimate of the average slope efficiency. To verify this, we re-analyzed each device’s light–current curve by extracting the slope efficiency from the linear region well below the onset of thermal rollover (see Supplementary Fig. S1). The extracted values cluster tightly around 0.15 W/A, with only minor variation with waveguide width and no systematic dependence on cavity length, confirming that the 0.152 W/A quoted here is representative of the entire device set. Detailed slope-efficiency data for different waveguide widths and lengths are provided in the Supplementary Information. As shown in the inset, a maximum single-facet GS power of 102 mW and a wall-plug efficiency (WPE) of 27.2% were achieved on a device with an 1850 × 6 μm^2^ cavity, assuming equal output from both facets. Suitable dielectric coating and facet passivation can further increase the output power of lasers. Figure 1d shows a representative light-current-voltage (L-I-V) characteristic of a 2100 × 3 μm^2^ laser at 20 °C and its spectrum at a bias of 3 × *I*_th_, the operating condition for the subsequent feedback studies. The threshold current is approximately 14.5 mA and saturation occurs at 310 mA, which is 21 times the GS threshold—well beyond the onset of ES lasing. This large ES-to-GS lasing threshold ratio is a recognized merit for robust feedback stability^19,20^.

To evaluate the *α*_*H*_, we monitored the net modal gain and lasing wavelength shift versus the normalized gain current based on sub-threshold ASE spectra^25^. Figure 1e shows *α*_*H*_ as a function of the wavelength. At the gain peak (~1275 nm), the *α*_*H*_ value is ~0.65, which is comparable to the state-of-the-art QD lasers reported and far exceeds that of the QW lasers (typically 2–5). The net modal gain ${g}_{{net},{modal}}$ is defined as the modal material gain ${g}_{{modal}}$ minus the waveguide propagation loss ${\alpha }_{{propagation}}$. The inset illustrates the net modal gain spectra under various sub-threshold biases, showing a gain of ~4.5 cm⁻¹ at the threshold. Here, ${g}_{{modal}}-{\alpha }_{{propagation}}$ approximately equals the mirror loss ${\alpha }_{{mirror}}$ at threshold, ensuring net zero round-trip gain. ${\alpha }_{{mirror}}$ of ~5.4 cm⁻¹ (indicated by the dashed line marking the threshold condition) is computed as $\frac{1}{L}\log \left(R\right)$, while laser length *L* = 2100 μm, laser facet reflectivity *R* = 32%. A similar net-modal-gain values near threshold are routinely reported for cleaved QD ridge lasers in^26^.

Figure 1f depicts the measured RIN spectra at 3 × *I*_th_ bias for two different power levels. At −2.3 dBm, the RIN reaches −150 dB/Hz and remains nearly flat, indicating a heavily overdamped response due to a large damping factor. It is important to note that the RIN is limited by the thermal noise of the photodiode due to the insufficient output power. As the output power increases to 4.2 dBm, the RIN further decreases to −160 dB/Hz, and a relaxation oscillation frequency (*f*_ROF_) of approximately 2.8 GHz emerges faintly from the noise floor. These results confirm that the laser is heavily damped, contributing to its inherent feedback tolerance. According to our and others previously published results, RIN of QD DFB is ~ −150 dB/Hz at 10 GHz^12^. RIN of QW DFB is >−150 dB/Hz at 10 GHz and exhibits significant relaxation oscillation^12^. For QD FP and QW FP, the minimum RIN of lasers are around −140 dB/Hz at 10 GHz^27^. The results are summarized as Table 1. Overall, the RIN of our QD FP lasers is comparable to—or better than—that of state-of-the-art QD FP lasers, and exceeds that of both QW FP and QW DFB lasers, confirming the superior noise characteristics of the QD gain medium.

**Table 1:** Measured RIN of QD FP compared to previous QD DFB, QW FP, and QW DFB

Laser	RIN value at 10 GHz	Relaxation oscillation peak	Optical power	Reference
QD DFB	−150 dB/Hz	No	0 dBm	^12^
QW DFB	>−150 dB/Hz	Yes	0 dBm	^12^
QD FP	−140 dB/Hz	No	-1.55 dBm	^27^
QW FP	−140 dB/Hz	Yes	-1.55 dBm	^27^
QD FP	−150 dB/Hz	No	-2.3 dBm	This work
	−160 dB/Hz		4.2 dBm	

### Optical feedback study

Optical feedback strongly influences semiconductor lasers dynamics by modulating both photon and carrier densities within the cavity. When feedback light re-enters the cavity, it perturbs the optical gain, and alters the refractive index through the *α*_*H*_ factor, inducing wavelength shift. A smaller *α*_*H*_ factor effectively decouples frequency fluctuations from gain variations, thereby increasing stability against feedback-induced noise. Similarly, a higher damping factor, which suppresses relaxation oscillations, further enhances feedback tolerance by minimizing carrier-photon oscillations and gain fluctuations.

This study considers two main categories of optical feedback. Incoherent optical feedback serves as an external noise source, primarily introducing intensity fluctuations that interact with carriers. These fluctuations, through the *α*_*H*_ factor, can translate into phase noise and ultimately degrade the laser coherence. In contrast, while coherent feedback can also induce phase fluctuations, these effects become negligible in the long-cavity feedback regime investigated here.

A way for estimating the critical feedback level *r*_crit_ for CC is described as follows^28^:

1 $${r}_{crit}=\frac{{\tau }_{L}^{2}{{\varGamma }_{R}}^{2}}{16{C}^{2}}\left(\frac{1+{{\alpha }_{H}}^{2}}{{{\alpha }_{H}}^{4}}\right)$$

Here, *τ*_L_ represents the photon cavity roundtrip time, ${\Gamma }_{R}$ denotes the damping factor, and *C* is the cavity coupling factor, which depends on the facet reflectivity *R* and is equal to $1-R/2\sqrt{R}$ for FP lasers. A low *α*_*H*_ factor and a high damping factor in QD lasers collectively elevates the feedback threshold, enabling stable operation under more stringent feedback conditions.

### A. Static optical feedback

**Fig. 2:**
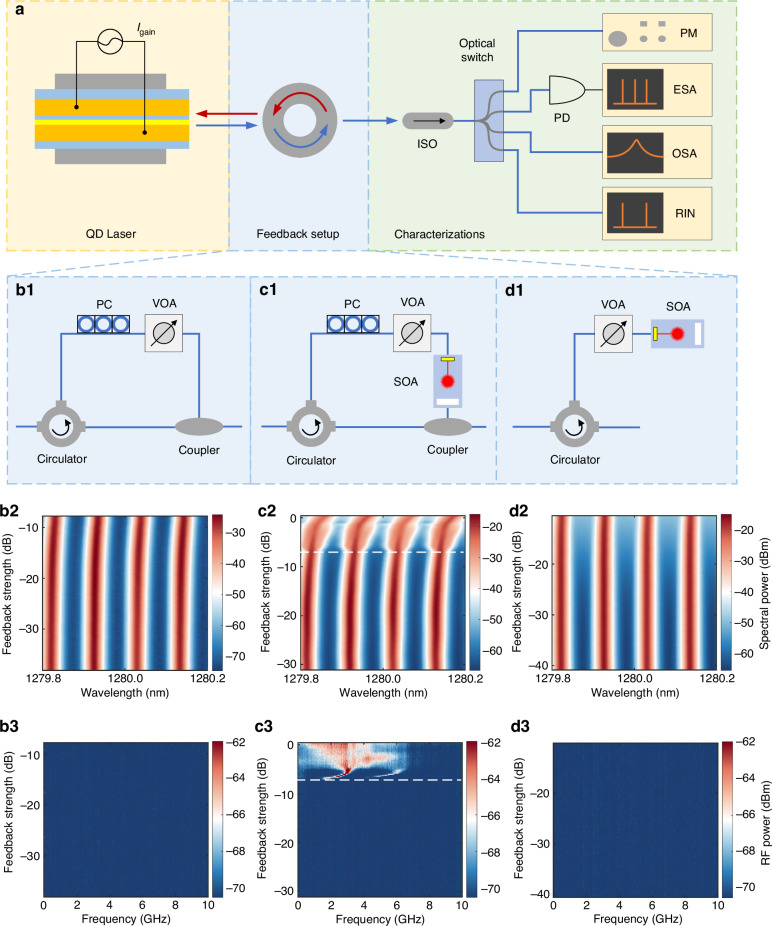
Static optical feedback measurement and corresponding optical and RF spectral mappings. **a** Schematic of the whole measurement setup. **b** Coherent optical feedback without SOA. **c** Coherent optical feedback with SOA. **d** Incoherent optical feedback. ISO optical isolator, PM optical power meter, PD photodetector, ESA electrical spectrum analyzer, OSA optical spectrum analyzer, VOA variable optical attenuator, PC polarization controller, SOA semiconductor optical amplifier

The experimental setup to characterize coherent optical feedback is schematically illustrated in Fig. 2a (see “Methods”). The experimental setup based on back reflector can only achieve a maximum feedback level of around −10 dB, which is insufficient to reach the critical threshold. To address this limitation, we incorporated a SOA into the feedback loop, enabling the feedback level to approach 0 dB. The experimental configurations for coherent feedback without an SOA, coherent feedback with an SOA, and incoherent feedback, are presented in Fig. 2b1, c1, d1, respectively.

Figure 2(b2), (b3) records the evolution of the four central longitudinal FP modes and RF spectra under coherent feedback without an SOA. The maximum achievable feedback in this case is −7.6 dB (17%). The FP modes remain stable with only a slight red shift as feedback strength increases, and no nonlinear oscillations appear in the RF spectrum. By incorporating SOA, the feedback setup achieves a maximum feedback value exceeding 0 dB (Fig. 2c2, c3), the FP modes remain stable until *r*_crit_ = −6.7 dB (21.4%), beyond which the system enters the CC regime. At this threshold, a slight red shift is observed, and the longitudinal FP modes broaden as CC sets in. Representative RF spectra reveal the evolution of laser dynamics under feedback: as the feedback approaches the threshold, periodic oscillations at the relaxation oscillation frequency and its harmonics become visible, followed by spectral broadening and the appearance of chaotic oscillations as coherence collapse occurs (Fig. S2).

For incoherent feedback measurements, the injected ASE light was generated internally by the SOA operating under the same bias current, without external input. Figure 2d2, d3 records the corresponding optical and RF spectral mappings. Even at −10.35 dB (9.22%), which is the maximum achievable feedback value for this configuration, the lasing wavelength remains unchanged and no nonlinear oscillations in the RF spectrum are detected, although the resonance trough in the spectrum rises due to increased ASE noise.

**Fig. 3:**
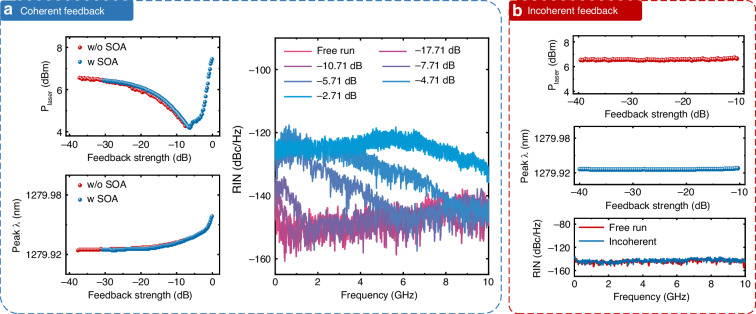
Laser power, wavelength shift, and RIN evolution under optical feedback. **a** Laser emitted power (*P*_laser_), peak wavelength shift (Peak *λ*), and RIN as a function of coherent optical feedback strength. **b** Corresponding measurements under incoherent optical feedback

Laser emitted power (P_laser_), peak wavelength shift (Peak λ), RIN under both coherent and incoherent optical feedback conditions are shown in Fig. 3a, b, respectively. For coherent feedback, as feedback increases to the critical threshold of −6.7 dB, the laser power decreases by ~2.5 dB. Beyond this critical point in the CC region, the laser power rapidly increases. Notably, the power and peak wavelength shift trends are nearly identical with and without the SOA, confirming the accuracy of the critical feedback point determined using the improved experimental setup. For incoherent optical feedback, even at the highest feedback intensity (−10.35 dB), the laser power and the wavelength peak remain essentially unchanged.

For RIN measurements, the input conditions were kept consistent to ensure a valid baseline comparison. For coherent feedback below critical feedback of −6.7 dB, the RIN remains smooth, except for a gradually increasing low frequencies noise component. After entering CC, the RIN level climbs significantly. In contrast, incoherent feedback has a negligible effect on RIN. The measured reinjected-light spectrum shows that the optical signal-to-noise ratio (OSNR) of the feedback path exceeds 36 dB for all feedback levels (Fig. S3), confirming that the coherent component of the returned field dominates over ASE by more than three orders of magnitude and explaining the negligible effect of incoherent feedback observed in Figs. 2d, 3b. Since RIN directly influences the performance of external intensity modulation schemes, the weak impact of coherent light on RIN is an important observational point that will be reinforced by subsequent transmission experiments.

**Fig. 4:**
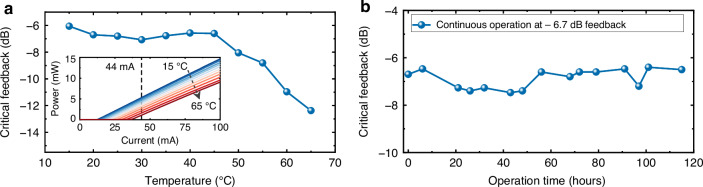
Temperature dependence and long-term stability of the critical feedback strength. **a** Measured critical feedback strength at various temperatures. The inset shows temperature-dependent single facet power (LI) curves with 2100 × 3 μm^2^ cavity size, the dashed lines represent the operation current (3 × *I*_th_ at 20 °C) in the feedback experiment. **b** Measured critical feedback strength during continuous operation near the feedback limit

We evaluated the reliability of the measured critical feedback point with respect to temperature variation and long-term stability, as summarized in Fig. 4. In Fig. 4a, the critical feedback strength is plotted at various stage temperatures. The inset shows LI curves of the same device (2100 × 3 µm² cavity), with the dashed line marking the operating current of 44 mA, three times the threshold current at 20 °C. Cooling the device to 15 °C increases the tolerance to nearly –6 dB, while between 20 and 45 °C the variation remains within only about 0.4 dB (±0.5 dB across 15–20 °C). Above 45 °C, however, the tolerance decreases rapidly. This trend can be attributed to the variations in the margin above threshold. At lower temperatures, the device operates far above threshold and is less sensitive to perturbation. At higher temperatures, however, the rising threshold current, carrier leakage and thermal escape reduce this margin, making the laser field more vulnerable to phase perturbations.

Figure 4b presents long-term measurements near the feedback limit. Under continuous operation with –6.7 dB feedback, the measured critical feedback point remained within a narrow range (–6.4 to –7.2 dB) across multiple time intervals up to 115 h. This small fluctuation indicates stable feedback tolerance across both a broad temperature range and extended operation.

To further evaluate reproducibility, three additional devices of the same geometry (2100 µm × 3 µm) were tested, yielding critical feedback thresholds of –6.7, –6.4, and –6.9 dB. The ~±0.3 dB variation indicates that the reported –6.7 dB threshold is representative rather than exceptional. Additional results for devices with different cavity lengths are provided in the Supplementary Information (Fig. S4).

For this part, we have demonstrated a QD FP laser with a critical feedback threshold as high as −6.7 dB. This remarkable performance can be attributed to several factors. First, optimizing the inhomogeneous broadening during the epitaxial growth efficiently enhanced the material gain and reduced the *α*_*H*_ factor. Second, a large damping factor effectively suppressed relaxation oscillations, while operation in long cavities provided an extended photon roundtrip time that further stabilized the device. Lastly, an exceptionally high ES-to-GS lasing threshold ratio of 21 ensured the laser’s outstanding stability. Collectively, these attributes contribute to the high feedback tolerance of QD lasers in this study. In addition, we have shown that the feedback tolerance remains robust across a broad temperature range of 15–45 °C and exhibits excellent persistence even after 115 h near the feedback limit.

### B. Transmission performance under optical feedback

**Fig. 5:**
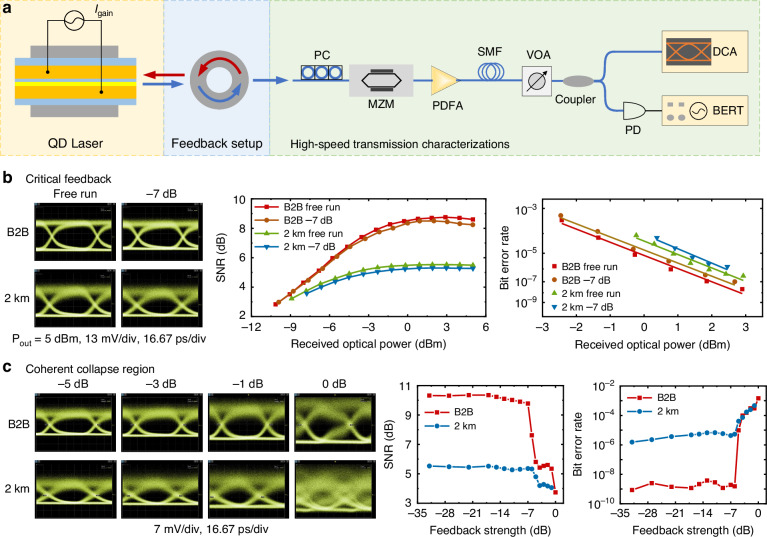
Transmission measurements under optical feedback. **a** Experimental setup. MZM Mach–Zehnder modulator, PDFA praseodymium-doped fiber amplifier, SMF single mode fiber, PD photodetector, DCA digital communication analyzer, BERT bit error rate tester. Eye diagrams, SNR, and BER performance (**b**) at the critical feedback level and (**c**) after entering the CC region

To evaluate the transmission performance under different feedback strengths, we conducted external modulation experiments at different feedback levels using the SOA-enhanced feedback loop. The experimental setup is shown on Fig. 5a (see “Methods”). We first studied the transmission performance just above the critical optical feedback of −7 dB strength. Figure 5b depicts eye diagrams, corresponding SNR and BER measured under the back-to-back (B2B) configuration and 2 km transmission conditions, both with and without feedback. The received optical power (ROP, P_out_) for all eye diagrams were kept constant. While a slight reduction in eye opening is observed after 2 km due to the chromatic dispersion and noise, the degradation caused by feedback in both links is almost negligible. This is confirmed by the nearly identical SNR and BER values under feedback and free-running conditions. After 2-km transmission, the BER power penalty is ~1 dB, indicating that feedback at this level does not significantly degrade transmission performance.

To further quantify the impact of feedback on system performance, we conducted a systematic set of BER-versus-ROP measurements under multiple discrete feedback levels, as presented in Supplementary Fig. 6 and Supplementary Table 1. These measurements show that the receiver-sensitivity penalty remains below 0.4 dB across all weak-to-moderate feedback levels. In addition, a dispersion analysis based on the measured RMS spectral width is provided in Supplementary Fig. 7. This analysis indicates that 0.5–1 dB of the 2 km penalty originates from chromatic dispersion, while only 0.3–0.4 dB near the CC threshold is attributable to optical feedback. These results are fully consistent with the back-to-back measurements and confirm that dispersion dominates the long-haul penalty, with feedback contributing only a minor additional degradation.

We further investigated the transmission performance as the feedback strength progressed from the onset of periodic oscillations into the full coherence collapse regime (Fig. 5c). As feedback increases beyond the CC threshold, the BER begins to rise, and the increase in output power is due to the feedback-induced power enhancement. Nevertheless, the eye diagram remains open and the BER stays below the 7% hard-decision forward error correction (HD-FEC) threshold (BER = 3.8 × 10⁻³) until the feedback strength reaches 0 dB, at which point the eye diagram becomes completely distorted. It is crucial to note that, due to equipment limitations, the amplified signal was not filtered. As a result, residual ASE noise from the PDFA and the broad spectral emission of QD laser, as observed through the fiber links, contribute to a “saturation” effect in SNR and BER measurements. This saturation behavior is independent of feedback effects and should be considered when interpreting the results.

It is worth noting that FP QD lasers were employed here to enable systematic exploration of feedback tolerance and ensure comparability with prior studies. Within this framework, our 10 Gbps transmission experiment over 2 km demonstrates that even near the critical feedback level (–7 dB), the penalty is negligible, and open eye patterns are preserved even at ~0 dB feedback. This penalty-free transmission near threshold provides direct evidence of the strong feedback tolerance of QD lasers, addressing the central question of whether they can reliably operate without isolators. At the same time, the relatively broad spectra of FP devices limit their suitability for maximizing transmission reach, where chromatic dispersion becomes dominant. Narrow-linewidth DFB QD lasers will therefore be more appropriate for future work aimed at higher bitrates, longer distances, and advanced modulation formats such as PAM4 or coherent schemes.

### C. Theoretical modeling under optical feedback

Critical feedback levels are strongly dependent on the external cavity length. To investigate this, we performed numerical simulations to model the laser under feedback based on standard Lang-Kobayashi approach^29^. The equations for the electric field amplitude $A$, phase $\phi$, and the carrier density $N$ are:

2 $$\frac{{dA}}{{dt}}={g}_{0}{NA}-{\alpha }_{{abs}}A+\frac{\kappa }{{\tau }_{L}}A\left(t-\tau \right)\cos \theta \left(t\right)$$

3 $$\frac{d\phi }{{dt}}={\alpha }_{H}{g}_{0}N+\frac{\kappa }{{\tau }_{L}}\frac{A\left(t-\tau \right)}{A\left(t\right)}\sin \theta \left(t\right)$$

4 $$\frac{{dN}}{{dt}}=J-\frac{N}{{\tau }_{c}}-{g}_{0}N{A}^{2}$$

Here, ${g}_{0}$ is the differential gain, ${\alpha }_{H}$ is the linewidth enhancement factor, ${\alpha }_{{abs}}$ is the propagation loss, ${\tau }_{L}$ is the roundtrip time in the laser cavity, $J$ is the pumping term, and ${\tau }_{c}$ is the carrier lifetime. The optical feedback is described by three parameters $\kappa$, $\tau$ and *θ*(*t*): $\kappa =\frac{1-R}{\,\sqrt{R}}\eta$ is the feedback strength, with $R$ the laser facet reflectivity and $\eta$ the total loss in the external cavity; $\tau$ is the roundtrip time in the external cavity; $\theta \left(t\right)$ is the phase of the optical feedback. The main goal of applying the LK model is to show the dependence of the coherence collapse boundary on the external cavity length. This simplified model assumes single-mode operation of the free-running laser and does not include incoherent ASE or the detailed carrier dynamics of quantum dot gain media, yet it remains effective for explaining the qualitative dependence of the feedback sensitivity on external cavity length. The ASE generated by the SOA behaves as white noise with a delta-function autocorrelation, making it uncorrelated with the intracavity field for any feedback delay and therefore unable to influence the cavity length dependence of the CC boundary. Consistent with this expectation, our measurements show that incoherent feedback has negligible impact on both the RIN and the CC threshold. The dominant dynamics are therefore governed by coherent reinjection, supporting the use of the Lang–Kobayashi framework for capturing the qualitative relationship between the CC boundary and external cavity length. To calculate the coherence collapse boundary, we performed a linear stability analysis of the above equations^30,31^. The laser field amplitude, phase, and carrier density are expanded into a sum of a stationary value and a small perturbation: $A={A}_{s}+\delta A$, $\phi =\,{\phi }_{s}+\delta \phi$, $N={N}_{s}+\delta N$. The perturbations are then Laplace-transformed, i.e., we set $\delta A=\delta {A}_{0}{e}^{\gamma t}$, $\delta \phi =\delta {\phi }_{0}{e}^{\gamma t}$, $\delta N=\delta {N}_{0}{e}^{\gamma t}$. The condition for the system of Eqs. (2–4) to have a non-zero solution is then given by^30^:

5 $$\begin{array}{l}{\gamma }^{3}+2{\gamma }^{2}\left(\frac{\kappa }{{\tau }_{L}\sqrt{1+{\alpha }_{H}^{2}}}-{\Gamma }_{R}\right)+\gamma \left({\omega }_{{ROF}}^{2}-\frac{4{\Gamma }_{R}\kappa }{{\tau }_{L}}+\frac{{K}_{1}{K}_{2}{\kappa }^{2}}{{\tau }_{L}^{2}}\right)\\+\left(\frac{{\omega }_{{ROF}}^{2}{K}_{2}\kappa \sqrt{1+{\alpha }_{H}^{2}}}{{\tau }_{L}}-\frac{2{\Gamma }_{R}{K}_{1}{K}_{2}{\kappa }^{2}}{{\tau }_{L}^{2}}\right)=0\end{array}$$

Where ${K}_{1}=1-{e}^{-\gamma \tau }$, ${K}_{2}=1+{e}^{-\gamma \tau }$, ${\omega }_{{ROF}}=2\pi {f}_{{ROF}}$ is the relaxation oscillation angular frequency, and ${\Gamma }_{R}$ is the damping factor of the laser. The parameter values used in Eq. (5) are summarized in Table 2. The laser remains stable as long all the solutions of Eq. (5) have a negative real part of $\gamma$. If a solution with a positive real part and a non-zero imaginary part emerges, the laser becomes unstable. To determine the coherence collapse boundary, we swept the feedback strength parameter $\kappa$ and identified the point where a solution with a positive real part appears.

**Table 2:** Calculation parameters for modeling the laser under feedback

Symbol	Physical meaning	Value	Unit	Determination method
${\tau }_{L}$	Roundtrip time	42	ps	Calculated from cavity length
${f}_{{ROF}}$	Relaxation oscillation frequency	2.8	GHz	From RIN measurement
${\alpha }_{H}$	Linewidth enhancement factor	0.65	None	From Linewidth enhancement factor measurement
$R$	Laser facet reflectivity	32%	None	From Fresnel equation calculation
${\Gamma }_{R}$	Damping factor	3.6	GHz	From RIN measurement

**Fig. 6:**
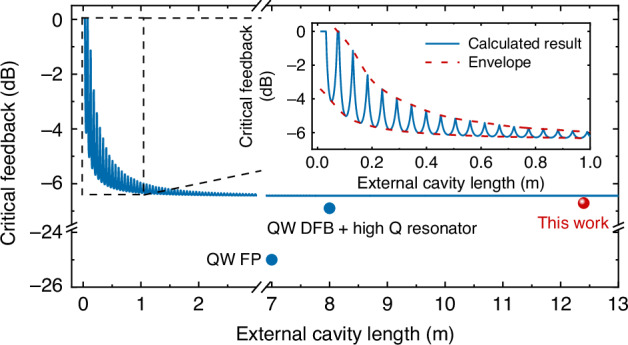
Modeled critical feedback strength versus external cavity length. Calculated critical feedback strength as a function of the external cavity length, showing the transition from the long-cavity regime to the short-cavity regime

The calculated dependence of the critical feedback level on the external cavity length is shown in Fig. 6. In the long-cavity regime, the coherence collapse boundary is independent of the cavity length, and the constant value aligns well with experimental results. Conversely, in the short-cavity regime, the critical feedback level oscillates as the cavity length varies, with a period inversely proportional to the relaxation oscillation frequency of the laser. Specifically, the distance between neighboring peaks is given by $\Delta L={v}_{g}/{f}_{{ROF}}$, where ${v}_{g}$ represents the group velocity of light propagation in the external cavity. Despite these oscillations, the curve is bounded by the two envelopes shown in red in the inset of Fig. 6. Notably, the minimum critical feedback strength in the short-cavity regime is consistent with that of the long-cavity regime. However, as the external cavity length decreases, the stability of the laser improves, with the coherence collapse boundary increasing to nearly 0 dB for centimeter-scale external cavities. These model predictions map directly onto practical layouts. Foundry-grade grating couplers used in LiDAR and sensing platforms typically return only –18 dB to –20 dB of Fresnel power^32,33^, giving at least a 12 dB safety margin with respect to the CC threshold even before any additional isolation. Moreover, most chip-level or chip-to-chip interconnects involve external-cavity lengths of 0.01–0.30 m^34,35^, precisely the region where Fig. 6 forecasts the CC boundary approaching 0 dB. Thus, QD lasers integrated in such environments can achieve a feedback tolerance far exceeding the −6.7 dB benchmark observed in the experiment. Compared to the previously reported results, the critical feedback value of QW FP lasers lies around –25 dB^7^ and can be increased to > –6.9 dB when a high-Q resonator is employed^8^, as also indicated in Fig. 6. This comparison highlights that, even in the most favorable resonator-enhanced QW case, the feedback tolerance remains below that of our standalone QD devices across all cavity-length regimes. Looking ahead, practical PICs may introduce additional effects such as thermal fluctuations, vibrations, and parasitic reflections. We have initiated exploration studies to address this, including thermal cycling, vibration testing, and integration trials on foundry platforms, which will provide the basis for a more comprehensive analysis in future work.

## Discussion

When determining the critical feedback level, both forward coupling losses and backward coupling losses must be considered, as they determine the actual power re-entering the laser cavity. Table 3 summarized the critical feedback thresholds of state-of-the-art reflection-insensitive lasers operating in long-cavity feedback regime, including DFB lasers, hybrid DFB + high-Q resonator structures, mode-locked lasers (MLL), and VCSEL systems. While some references only account for forward coupling loss and neglect the typical ~3 dB backward coupling loss, our device was evaluated under the stricter criterion of including both. Even so, it achieves a CC threshold of −6.7 dB, comparable to the best hybrid DFB + resonator platforms (>−6.9 dB), and substantially more tolerant than typical QW FP (≈−25 dB) and QW DFB (≈−31 dB) lasers, QD DFB lasers (−14 dB at 25 °C, −8 dB at 85 °C), QD MLLs (>−10 dB), QDash FP lasers (−23 dB), and VCSELs (≈−29 dB) used in short-reach isolator-free links. Taken together, these comparisons confirm that our device maintains superior reflection insensitivity and achieves best-in-class feedback tolerance among isolator-free laser solutions at similar wavelengths.

**Table 3:** Critical feedback level comparison for the state-of-the-art reflection-insensitive laser

Platform	Wavelength	Type	Critical feedback level	Backward coupling loss	Ref. No.
QD	1.28 μm	FP	−6.7 dB	Considered	This work
QD	1.3 μm	FP	>−7.4 dB	Not Considered	^7^
QD	1.3 μm	FP	>−8 dB	Not Considered	^18^
QD	1.3 μm	FP	>−13 dB	/	^15^
QD	1.3 μm	FP	>−10 dB	/	^21^
QD	1.3 μm	FP	−8 dB	/	^38^
QD	1.3 μm	DFB	−14 dB at 25 °C −8 dB at 85 °C	/	^39^
QD	1.3 μm	MLL	>−10 dB	Not considered	^22^
QDash	1.31 μm	FP	−23 dB	Not considered	^40^
QW	1.55 μm	FP	−25 dB	Not considered	^7^
QW	1.24 μm	VCSEL	−29 dB	Not considered	^41^
QW	1.55 μm	DFB	−31 dB	/	^6^
QW	1.55 μm	DFB+ high Q resonator	>−6.9 dB	Considered	^8^

“/” indicates ambiguity

In conclusion, this work shows that QD lasers sustain stable telecom-grade performance under feedback levels where QW devices lose stability, often tens of decibels earlier. Using a setup that reaches 0 dB and an in-loop SOA to overcome passive loss, we directly observed CC at −6.7 dB (21.4% return). Near this limit, devices maintained penalty-free 10 Gbps transmission under external modulation, open eyes up to near 0 dB, stable operation across 15–45 °C with ±0.5 dB drift, more than 100 h of continuous testing, and reproducibility of ~±0.3 dB. RIN and RF spectra documented the transition into collapse. Modeling supports these findings and predicts that centimeter-scale external cavities typical of PIC layouts shift the CC boundary closer to 0 dB, confirming that QD lasers are most tolerant under the conditions where they will actually be used. Benchmarking establishes the reported QD lasers as competitive with advanced hybrid designs and superior to other common sources. Beyond performance, removing isolators simplifies packaging and improves manufacturability, paving the way for reliable, energy-efficient photonic integration. Ongoing studies on environmental stress and foundry-scale deployment will extend this foundation toward practical system adoption.

## Materials and methods

### QD laser fabrication

The QD laser structure was grown on a (001) GaAs wafer using a Veeco Gen-II solid source molecular beam epitaxy (MBE) system. A GaAs/Al_0.4_Ga_0.6_As graded-index separate confinement heterostructure was used as both the top and the bottom cladding layers. The active region consists of five layers of InAs QDs embedded in In_0.15_Ga_0.85_As QWs grown at 495 °C, followed by an indium flush at 580 °C after capping the QDs. Each barrier layer between the dot layers included a 10 nm GaAs region *p*-doped with Be at 5 × 10^17 ^cm^−3^ for high temperature performance^36^. Minimizing inhomogeneous broadening in the QD assembly is essential for increasing material gain and maintaining a low *α*_*H*_ factor across the operating range^16,37^. Such inhomogeneity, or the QD size variation in this case, is attributed to the random nature of the Stranski-Krastanov growth of the self-assembled QDs and the Oswald ripening process where the dots exchange materials between each other in the subsequent stage of the growth. To minimize the inhomogeneous broadening and enhance the material gain, we carefully tuned the InAs deposition rate and the arsenic overpressure to suppress crosstalk between the dots during the nucleation and the ripening process. Contrary to conventional assumptions, we found that absolute arsenic overpressure, rather than V/III ratio alone, was the controlling factor of QD quality. By fixing the arsenic overpressure at 1 × 10^−6^ torr and slowly adjusting the indium flux, we achieved uniform, high-quality QDs with a strong, narrow ground-state emission centered around 1280 nm and a full-width at half maximum (FWHM) of ~28 meV at room temperature.

### Static optical feedback characterizations

Figure 2a depicts the experimental setup for characterizing optical feedback. The QD laser, biased at 3 × *I*_th_, was mounted on a thermoelectric cooling stage to maintain stable operation at a temperature of 20 °C. In our optimized passive configuration (Fig. 2b1), we improved chip-to-fiber coupling and replaced the standard 50:50 coupler with a 90:10 device, yet the maximum round-trip feedback remains capped at ~−7.6 dB due to residual insertion losses in the fiber loop (circulator, connectors, polarization controller). The SOA provides up to 10–15 dB of in-loop gain, compensating these remaining losses and bridging the ~0.9 dB gap required to observe the −6.7 dB coherence-collapse threshold. For the coherent feedback, the emission from the QD laser was first coupled into a lensed fiber and then routed through a three-port fiber-optic circulator. The output was split using an optical coupler into two paths: one for feedback and one for the output path. In the feedback path, a VOA controlled the feedback strength, and a manual polarization controller aligned the polarization direction with the laser’s transverse electric mode before re-injection into the cavity. The feedback strength is defined as the ratio between the returned power to the free-space emitted power at the coupling facet, accounting for both the forward and backward coupling loss. To ensure accurate feedback characterization, we performed a per-component loss calibration using a calibrated power meter and an integrating sphere. First, we measured the sphere’s current–power response, then recorded the total on-chip output versus the fiber-coupled power to extract a one-way chip-to-fiber loss of 2.2 dB (–4.4 dB round-trip). Next, with the VOA set to a known attenuation, we measured the combined loss of the circulator, coupler, and polarization controller by passing the laser. Finally, by scanning the VOA attenuation, we directly obtained the net on-chip feedback level plotted on the horizontal axis. With the SOA in place, the external cavity lengths in the feedback loop with SOA were approximately 12.4 m, corresponding to a resonance frequency of ~8 MHz. These frequencies are significantly lower than the laser’s *f*_ROF_, positioning the system in a long-delay regime where phase effects are negligible. The remaining output power, isolated from the feedback path, was utilized for characterizations including laser power evolution, optical and electrical spectra, and RIN measurements.

### Transmission experiment under optical feedback

External modulation experiments at different feedback levels were conducted using the SOA-enhanced feedback loop. The experimental setup is shown on Fig. 5a. A 10 Gbps NRZ signal was generated from a bit error rate tester with a pseudo-random binary sequence (PRBS) length of 2^15^-1. The QD laser output under feedback was sent to a 30 GHz Mach–Zehnder modulator. A polarization controller was employed to ensure maximum coupling efficiency. The modulated signal was amplified using a praseodymium-doped fiber amplifier and then transmitted through a 2 km standard single-mode fiber. After passing through a VOA for power adjustment, the signal was split into 2 paths: one path was directly sent to a digital communication analyzer to record the eye diagram, and the other was detected by a 70 GHz bandwidth photodetector for bit error rate measurements.

## Supplementary information

Supplementary Information

## Data Availability

All data generated or analyzed during this study are available within the paper and its Supplementary Information. Further source data will be made available upon reasonable request.
