# Supplementary material for: Exploring the feedback limits of quantum dot lasers for isolator-free photonic integrated circuits
Source: Light Sci Appl. 2026 Jan 30;15:96. doi: 10.1038/s41377-026-02185-w (PMC12855847; doi:10.1038/s41377-026-02185-w)
Supplement: Supplementary file 1 — Supplementary Information [file 41377_2026_2185_MOESM1_ESM.pdf]

Supplementary Information for:

Exploring the feedback limits of quantum dot lasers for isolator-free photonic integrated circuits

YING SHI,[1,†] BOZHANG DONG,[1,†] XIANGPENG OU,[1,†] ARTEM PROKOSHIN,[1] CHEN SHANG,[2] JOHN E. BOWERS,[2] AND YATING WAN[1,*]

[1]*Integrated Photonics Lab, King Abdullah University of Science and Technology (KAUST), Thuwal, 23955-6900, Kingdom of Saudi Arabia*

[2]*Institute for Energy Efficiency, University of California Santa Barbara, Santa Barbara, CA 93106, USA*

[†]*These authors contributed equally*

**yating.wan@kaust.edu.sa*

1. Slope efficiency for all lasers

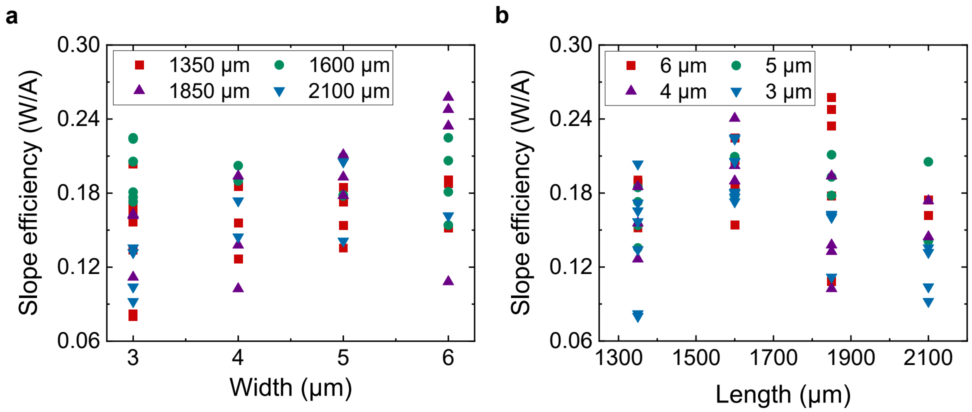

Fig. S1. Slope efficiency for all lasers with (a) width and (b) length as independent variables.

Figure S1. shows the slope efficiency for all lasers with width and length as independent variables. The waveguide width has only a minor influence on slope efficiency, and no systematic trend with cavity length is observed. The individual slope efficiencies cluster tightly around 0.15 W/A, confirming that the "best-fit" value of 0.152 W/A quoted in the main text is indeed a conservative average representative of our device set.

2. RF spectra under different feedback conditions

The traces in Figure S2 are extracted from the mapping plot in Fig. 2(c3) of the main text, highlighting representative feedback regimes. At the onset of instabilities, periodic oscillations emerge, driven by undamped relaxation oscillations, with the relaxation oscillation frequency (ROF) and its higher harmonics clearly observed. With further increase in feedback strength, the system evolves into the coherence collapse regime, where the laser output becomes dominated by broadband chaotic oscillations.

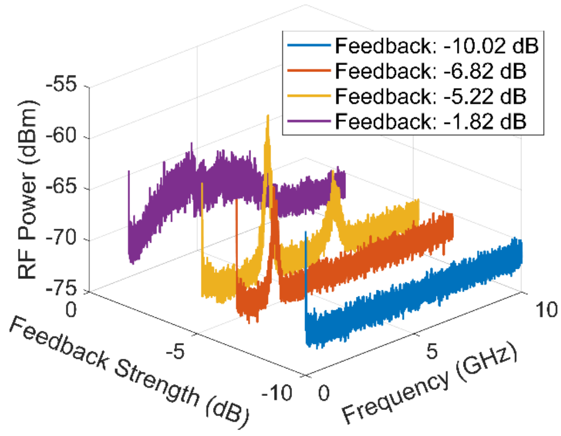

Fig. S2. Representative RF spectra extracted from Fig. 2(c3) of the main text.

3. Mixed coherent and incoherent feedback: OSNR and experimental impact

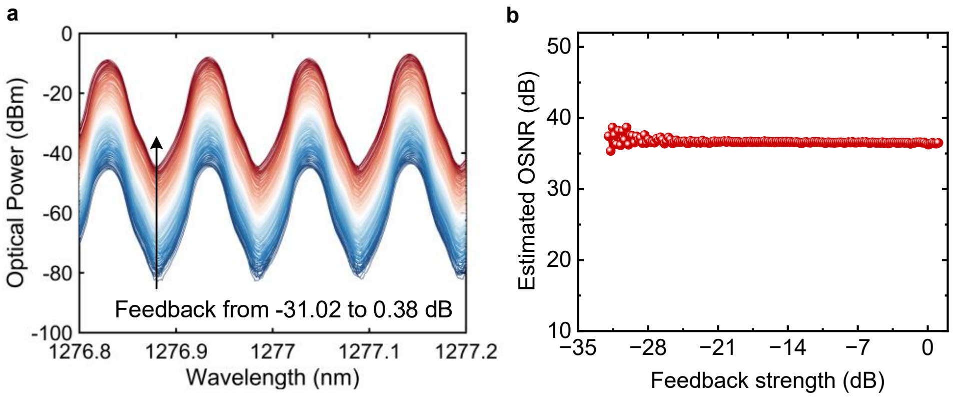

Fig. S3. (a) The spectrum of feedback light after SOA amplification and (b) the corresponding estimated OSNR.

Supplementary Figure 3(a) shows the measured spectrum of the reinjected light after SOA amplification. The optical signal-to-noise ratio (OSNR) in Supplementary Figure 3(b), estimated from the ratio between the longitudinal-mode peak and the intermodal valley, exceeds 36 dB for all feedback levels. This indicates that the coherent component of the returned field dominates over the ASE contribution by more than three orders of magnitude. Purely incoherent feedback generated by ASE from the SOA was also examined, as shown in Fig. 2(d2), Fig. 2(d3), and Fig. 3(b) of the main text. The lasing wavelength, optical spectra, and RIN remain essentially unchanged even at the maximum incoherent feedback of -10.35 dB, confirming that incoherent ASE has negligible influence on the laser dynamics and does not affect the measured coherence collapse threshold. Since the ASE introduced by the SOA is white noise with a delta-function autocorrelation, it remains uncorrelated with the intracavity field for any external cavity length. The dominant dynamics are therefore governed by the coherent reinjected field, consistent with the qualitative cavity-length dependence described by the Lang–Kobayashi model in the main text.

4. Measured critical feedback strength with different cavity lengths

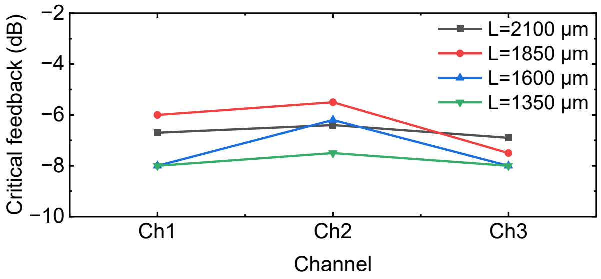

Fig. S4. Measured critical feedback strength with different cavity lengths.

As shown in Eq. (1) of the main text, the critical feedback (CC) threshold is directly related to the laser cavity length (photon cavity roundtrip time), linewidth enhancement factor, and damping factor. To assess reproducibility, we tested multiple devices with the same dimensions as those reported in the manuscript (2100 μm×3 μm). All exhibited CC thresholds consistently around –6.7 dB, with device-to-device variation of only ~±0.3 dB. This confirms that the reported –6.7 dB value is representative of this device geometry. We also measured devices with different cavity lengths (1350–2100 μm) and a fixed width of 3 μm. As summarized in Fig. S4, the CC threshold ranged from –6.4 to –6.9 dB for L = 2100 μm (centered at –6.7 dB),

–5.5 to –7.5 dB for L = 1850 μm, –6.2 to –8.0 dB for L = 1600 μm, and –7.5 to –8.0 dB for L = 1350 μm.

Despite the limited sample size, the results reveal a clear cavity-length dependence of the CC threshold, consistent with the theoretical model. To establish more comprehensive statistics, we will investigate larger sets of devices fabricated with improved process uniformity (e.g., tighter control of threshold currents).

5. Measured operating voltage under optical feedback

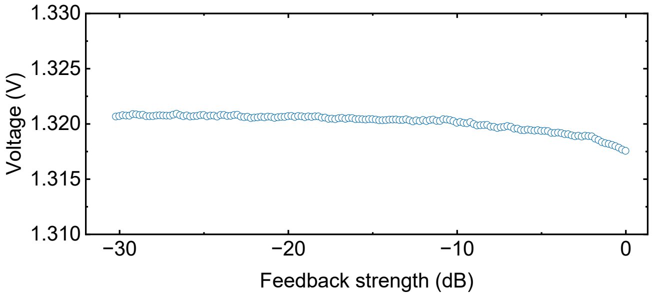

Fig. S5. Measured operating voltage at different feedback levels under a fixed bias current of 44 mA.

We investigated the electrical characteristics of our device across the full optical feedback as Fig. S5. At a fixed injection current of 44 mA, the operating voltage decreases slightly but systematically as feedback increases from –33 dB to 0 dB (from ~1.321 V to ~1.317 V). This trend can be explained by feedback-induced reduction in threshold carrier density, leading to lower junction voltage and marginally reduced electrical power consumption. In practical terms, this indicates that optical feedback not only affects the laser's optical dynamics but also reduces its thermal load, leading to improved operating stability in isolator-free PICs.

6. Systematic BER-versus-ROP measurements under multiple discrete feedback levels

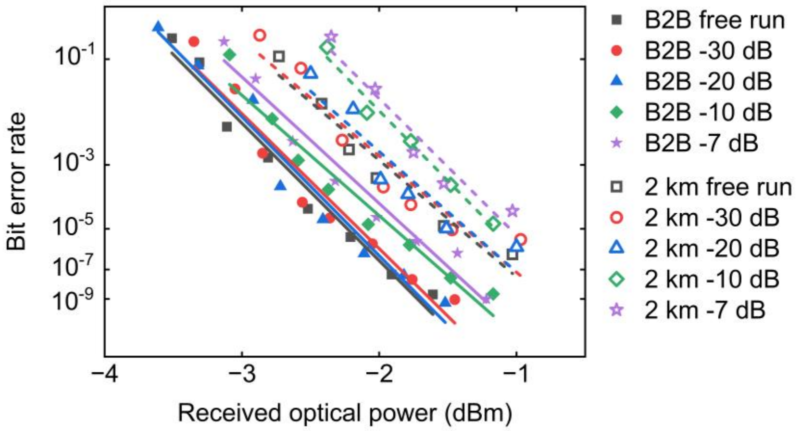

Fig. S6. BER-versus-ROP curves measured under feedback levels of free-running, –30 dB, –20 dB, –10 dB, and –7 dB. The curves show minimal variation at low and moderate feedback levels and only a slight sensitivity shift near the critical –7 dB point.

Supplementary Table 1. Receiver sensitivity and corresponding power penalty extracted from B2B measurements.

Feedback level (dB)	Receiver sensitivity (dBm)	Power penalty (dB)
Free run	-2.7	
-30	-2.6	0.1
-20	-2.6	0.1
-10	-2.4	0.3
-7	-2.3	0.4

To obtain a comprehensive and quantitative assessment of the transmission performance under controlled optical feedback, we conducted a systematic set of BER-versus-received-optical-power (ROP) measurements across multiple discrete feedback levels. These measurements complement the main-text results obtained at the free-running and –7 dB feedback points and enable a more detailed evaluation of how feedback influences receiver sensitivity. Experiments were carried out under a back-to-back (B2B) and 2-km transmission configuration at feedback strengths of free-running, –30 dB, –20 dB, –10 dB, and –7 dB. For each condition, the receiver sensitivity was determined at the HD-FEC threshold (BER = 3.8×10^{-3}), and the corresponding power penalty was calculated relative to the free-running baseline.

Supplementary Fig. 6 presents the measured BER-versus-ROP characteristics. The BER curves at –30 dB and –20 dB overlaps closely with the free-running case, indicating that weak feedback introduces negligible degradation in receiver sensitivity. As the feedback strength increases, a gradual shift in sensitivity is observed; however, even near the onset of coherence collapse at –7 dB, the penalty remains modest at approximately 0.4 dB. A summary of the receiver sensitivities and power penalties extracted from B2B measurements is provided in Supplementary Table 1. The tabulated values quantitatively illustrate the minimal impact of weak-to-moderate feedback and highlight the robustness of QD lasers against feedback-induced transmission degradation. These results confirm that QD lasers maintain strong tolerance to moderate optical feedback, consistent with the behavior observed in the main text.

7. RMS spectral width and chromatic dispersion analysis

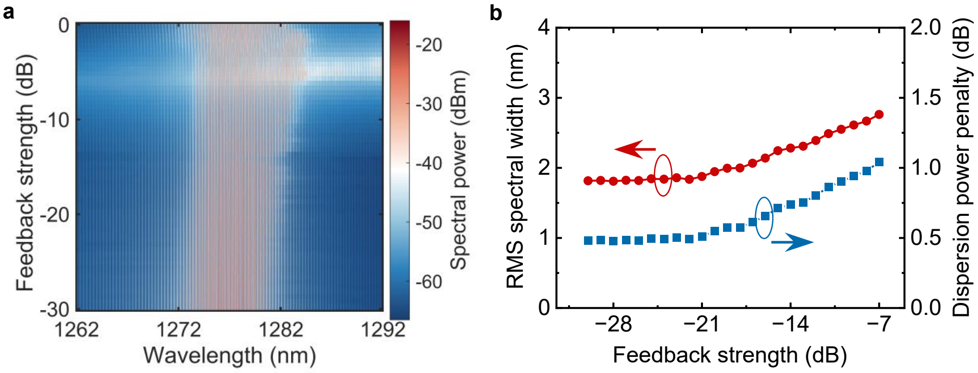

Fig. S7. (a) Optical spectral mapping under different feedback levels. (b) Calculated RMS spectral width and the corresponding dispersion-induced power penalty for a 10-Gbps NRZ signal over a 2-km transmission link.

To quantitatively distinguish the contributions of chromatic dispersion and optical feedback to the 10 Gbps, 2 km transmission performance, we measured the RMS spectral width of the laser under each feedback condition and used it to calculate the dispersion-induced power penalty

following standard IM-DD analysis. The RMS spectral width σ_λ (in nm) was obtained from the standard deviation of the optical power spectrum

$$\sigma_\lambda = \sqrt{\frac{\int (\lambda-\lambda_0)^2 S(\lambda) d\lambda}{\int S(\lambda) d\lambda}}, \lambda_0 = \frac{\int \lambda S(\lambda) d\lambda}{\int S(\lambda) d\lambda}$$

where $S(\lambda)$ is the power spectral density and λ_0 is the power-weighted center wavelength. The corresponding RMS pulse broadening for transmission through a fiber link of length L is given by

$$\Delta\tau = |D| L \sigma_\lambda$$

where D is the chromatic-dispersion parameter. The associated dispersion-induced power penalty for NRZ signaling can be estimated using the widely adopted eye-closure approximation [1],

$$Penalty_{disp} = 10\log_{10}\{1 + (\pi B \Delta\tau)^2\}$$

where B is the bit rate. In our experiment, we use $D \approx 3\ \mathrm{ps/(nm \cdot km)}$ at 1278 nm [1], $L = 2\ \mathrm{km}$, $B = 10\ \mathrm{Gbps}$.

Fig. S7(a) presents the measured optical spectra at different feedback levels. The longitudinal modes remain nearly unchanged up to the critical feedback point, and only a slight modification in the spectral envelope is observed. This small change produces a modest variation in the RMS spectral width and therefore in the dispersion-induced penalty. Fig. S7(b) shows the calculated RMS spectral width and the corresponding dispersion penalty for the 2 km link. Across the feedback range from -30 dB to -7 dB, the dispersion-induced penalty varies between 0.5 and 1 dB. These results can be compared directly with the experimentally measured total penalty in the 2 km transmission link, shown in Fig. 5(b) of the main text and Fig. S6, which lies between 1 and 1.3 dB. The calculated dispersion component of 0.5 to 1 dB therefore accounts for most of the observed degradation.

Indeed, the B2B configuration, where dispersion is absent, also shows that only the -10 dB and -7 dB feedback conditions introduce a measurable penalty of 0.3 to 0.4 dB, while weaker feedback levels cause negligible degradation. This matches the residual portion of the total 2 km penalty after subtracting the dispersion contribution.

Overall, the dispersion-induced penalty derived from the measured RMS spectral width agrees with both the total measured penalty in the 2 km link and the feedback-induced penalty extracted from the back-to-back measurements. This confirms that the system-level degradation in our 2-km transmission experiments is dominated by chromatic dispersion, with only a small additional contribution from optical feedback.

Reference

[1] Agrawal, G. P. (2012). Fiber-optic communication systems. John Wiley & Sons.
